# Self-Compassion Scale (SCS): Psychometric Properties of The French Translation and Its Relations with Psychological Well-Being, Affect and Depression

**DOI:** 10.1371/journal.pone.0152880

**Published:** 2016-04-14

**Authors:** Ilios Kotsou, Christophe Leys

**Affiliations:** Université Libre de Bruxelles, Brussels, Belgium; University of Akron, UNITED STATES

## Abstract

Over the past few years, the topic of self-compassion has attracted increasing attention from both scientific and clinical fields. The Self-Compassion Scale (SCS) was created to specifically capture this way of being kind and understanding towards oneself in moments of turmoil. In this article, we present a French adaptation of the SCS. We first explore the psychometric properties of this adaptation and then investigate its relation to psychological well-being. As in the original version of the SCS, the French adaptation has a strong 6-factor structure but a weaker hierarchical second order structure. However the bi-factor model yields a good omega index suggesting the relevance of a single score accounting for self-compassion. Moreover, there was a relation between the SCS and classical outcomes such as a positive relation with psychological well-being and negative relation with depressive symptoms. We then hypothesized that self-compassion would have a moderating role on the relation between affect and depression. This hypothesis was confirmed: expressing negative affect is correlated with depressive symptoms; however, being kind with oneself lowers depressive symptoms even when expressing negative affect. In conclusion, this research presents a valid self-compassion measure for French-speaking researchers and clinicians and outlines the need for further research on the concept of self-compassion.

## Introduction

“The worst loneliness is to not be comfortable with yourself.”

*Mark Twain*.

Most human beings, all across the world, desire happiness for their children or loved ones [1]. Treating a good friend –or even a stranger- with kindness and caring is common. Paradoxically, it is also common to treat ourselves harshly, particularly in moments of failure and difficulty [2]. Our need for affiliation leads us to frequent social comparisons [3] that may trigger self-critical attitudes such as envy, guilt or regret [4]. Self-criticism has been associated with poorer mental health and increased risk of depression (Murphy et al., 2002). In contrast, self-compassion, the psychological construct that makes reference to the disposition to treat ourselves with the kindness and compassion we give to others, has been the subject of increased research over the last 10 years [5]. According to Neff [6], self-compassion is made of three components “1) extending kindness and understanding to oneself rather than harsh self-criticism and judgment; 2) seeing one’s experiences as part of the larger human experience rather than as separating and isolating; and 3) holding one’s painful thoughts and feelings in balanced awareness rather than over-identifying with them” (p.224). Kristin Neff developed a questionnaire to assess this construct [6] called the Self-Compassion Scale (SCS). The SCS is a 26-item self-reporting questionnaire with a 5-point Likert response format (from 1 = almost never to 5 = almost always). The original scale was meant to measure the three main components of self-compassion on separate subscales, but analyses revealed a structure of 6 inter-correlated factors: self-kindness (e.g., I’m kind to myself when I’m experiencing suffering), self-judgment (e.g., I’m disapproving and judgmental about my own flaws and inadequacies), common humanity (e.g., I try to see my failings as part of the human condition), isolation (e.g., When I fail at something that’s important to me, I tend to feel alone in my failure), mindfulness (e.g., When something painful happens I try to take a balanced view of the situation), and over-identification (e.g., When I fail at something important to me I become consumed by feelings of inadequacy). Recent work by Neff [7] suggests that the SCS can be used as an overall measure of self-compassion or as a measure of the six subscales, depending on the goals of the researchers.

Initial empirical work has shown that self-compassion is positively and significantly related to psychological health (e.g., happiness, optimism, positive affect, wisdom, personal initiative) beyond that which can be accounted for by personality. Self-compassion appears to have an impact on well-being as well for the general population [2], older adults [8] and adolescents [9]. Self-compassion may be a useful resource to protect caregivers from stress [10], and may be applied in different clinical settings to treat depression [11,12], shame [13] and social anxiety [14]. Self-compassion has been investigated as a resilience factor in situations of traumatic stress [15] or psychological vulnerability [16]. It may be seen as a buffer when facing distressing events [17]. Recent assessments of, intervention programs showing that self-compassion can be enhanced, with a meaningful impact on well-being [18].

On the whole, these results suggest that the SCS not only constitutes a useful way of measuring self-compassion, but that the construct has important implications on psychological health and well-being. We therefore decided to develop, test and validate a French version of the SCS in order to make it available to French-speaking clinicians and researchers.

Lastly, as a first application, we also aimed at investigating the possible moderating role of the SCS on the link between affect and depression. Indeed, previous studies investigating cognitive vulnerability models have demonstrated that maladaptive helplessness is involved in the development and maintenance of depression [19]. These vulnerability factors become activated by aversive life circumstances and negative emotions [20], and they may increase the occurrence of depression through their interaction with environmental stressors, [21]. The experience of negative emotions can lead to depression, but it would be interesting to explore under which conditions such negative affect influences depression. However, from a contextual behavioural perspective (in which self-compassion is conceptualised), it is not the negativity of the emotions, but rather how one responds to those emotions when they arise, that is critical to the onset of depression [22]. From this perspective, it makes sense to hypothesize that self-compassion may act as a protective factor that would buffer the impact of negative affect on depression. If this holds true, we predict a moderating effect of self-compassion on the effect on negative affect (H1). Conversely, feeling positive affect seems to protect against depression symptoms, or rather, failing to feel positive affect will be related to anhedonia [23], which is a main symptom of depression [24]. In line with Hayes et al. (2013)’s way of thinking, self-compassion may protect against such a mechanism. Hence, we predict a moderating effect of self-compassion on the link between positive affect and depression (H2).

## Method

### Participants

A sample of 1554 participants (1371 women, 183 men) was recruited through an announcement posted online. Participants were between 15–83 years old (mean age = 42.92 years, SD = 12.61). 64% of them had at least an undergraduate level of education.

### Materials

SCS. The SCS is a 26-item scale. The questionnaire starts with the following sentences: “Please read each statement carefully before answering. To the left of each item, indicate how often you behave in the stated manner, using the following scale.” Each statement (e.g.; I’m kind to myself when I’m experiencing suffering) is rated on a 5-point Likert scale ranging from 1 (almost never) to 5 (almost always). The French version of the SCS (see: S2 File. French version of the SCS questionnaire) was translated with a back- translation procedure. Discrepancies emerging from this back-translation were discussed, and adjustments to the translation were made. The SCS is designed to either assess the six subscales of self-compassion separately or else as a total self-compassion score. However, the SCS is usually used with a single score.

Mindful Attention Awareness Scale (MAAS). The MAAS [25] is a 15-item instrument measuring the general tendency to be attentive to, and aware of present-moment experience in daily life (e.g.; “I find myself doing things without paying attention”), using a 6-point Likert scale (“almost always” to “almost never”). It has a single-factor structure and yields a single total score (α = .88), validated in French [25].

The Satisfaction with Life Scale (SWLS). TheSWLS (Diener, Emmons, Larsen, & Griffin, 1985) assess life satisfaction. This validated five-item instrument assesses satisfaction with the respondent’s life as a whole (e.g., “I am satisfied with my life”). Participants indicate agreement or disagreement on a 7-point scale (1 = strongly agree, 7 = strongly disagree). It is a single factor concept (α = .89) validated in French [26].

Subjective Happiness Scale (SHS). Happiness was assessed using the SHS (Lyubomirsky & Lepper, 1999), whose French validation is in progress French (Kotsou & Leys, in prep.).The measure is composed of four items scored on a 7-point Likert scale (e.g., Item 1—“In general I consider myself”: 1 = Not a very happy person to 7 = A very happy person), and provides an overall score reflecting whether one is a happy or an unhappy person (α = .81).

Positive and Negative Affect Schedule (PANAS). The PANAS [27]. French validation) is an adjective checklist which which individuals use 5-point Likert scales to rate the degree to which they generally feel specific emotions (e.g., “Proud” for PA; “Nervous” for NA). The PANAS is a 20-item tool that measures two dimensions of mood: Positive Affect (PA, 10 items, α = .84) and Negative affect (NA, 10 items, α = .89).

Beck Depression Inventory Short Form (BDI-SF). The BDI-SF is an instrument used for assessing the severity of depression. It comprises 13 items (α = .86) rated on 4-point Likert scales (ranging from 0 to 3), with a global score that ranges from 0 to 39 (e.g.; “I feel discouraged about the future”). We used the French-validated version of the inventory for this study [28].

The Cronbach alpha values of each measure are presented in Table 1.

**Table 1:** Descriptive statistics of the measures used.

Variables	n	M	SD	Α
SCS	1554	2.88	.79	.94
MAAS	1554	3.63	.92	.88
SWLS	1554	4.41	1.49	.89
SHS	1554	4.49	1.32	.81
PA	1554	3.37	.65	.84
NA	1554	2.46	.69	.89
BDI	1554	6.77	5.71	.86

### Procedure

Ethical standards related to privacy, anonymity and informed consent, in accordance with the ethics code of American Psychological Association, were respected. Participants were recruited through an online questionnaire posted on the webpage of a TV show on happiness. Participants were voluntary, they were aware they were participating in a study and could withdraw from the questionnaire at any time. No identifying information (e.g. name) was requested, but participants could leave their email if they agreed to be contacted for further research. This information was kept under lock by the principal investigator. We consulted the university ethics committee about the study, and the committee waived the requirement for ethical approval (See S5 File. Retrospective waivers of approval). To avoid the risk of undue inducement [29], no compensation was given to participants.

## Results and Discussion

### Factorial Structure

We tested 3 different models in order to validate the scale: a six-factor model, a second-order hierarchical model (as presented in the initial validation study) and a bi-factor model. In a recent paper [7], Neff argues that a second-order hierarchical model is not the most effective way to determine the validity of the SCS scale, and that a bi-factor model including an omega index should be tested. An omega index is an estimation of the amount of variance that can be attributed to the overall factor, and is considered as an alternative and more effective measure than goodness of fit index alone of whether an overall score should be used. According to Neff (8), self-compassionate behaviour is more accurately represented as a construct resulting from the interactions of the attitudes one uses to respond to suffering (reflected by the different items of the scale) rather than being the sum of its six subscales. In that regard, a bi-factor model is a better way to represent this conceptualization of self-compassion because while a second order hierarchical model supposes that the target factor is explained by the correlation of the subscales factors, in a bi-factor model, the target and the group factors are directly related to the items and are not allowed to correlate between themselves.

Using Mplus software 6.11, we ran a confirmatory factor analysis (CFA) with the maximum likelihood (ML) on the variance-covariance matrix [30]. Goodness of fit was tested with χ^2^ (a non-significant value corresponds to an acceptable fit). Because χ^2^ are well known to increase with sample size and degree of freedom [31], we also examined other important indices that have a conventional cut-off. As recommended by Hu and Bentler [32], we used two other fit indices: The Standardized Root Mean Square Residual (SRMR) and the Root Mean Square Error of Approximation (RMSEA). The SRMR controls for the misspecification of the factor covariance and the RMSEA assesses the misspecification of the factor loadings. We also report the Comparative Fit Index (CFI) and the Tucker-Lewis Index (TLI), two widely used indices that also have conventional cut-off values [33]. An SRMR between 0 and .05 is an indication of a good fit, and one between .05 and .10 indicates an acceptable fit. An RMSEA between 0 and .05 is seen as an indicator of a very good fit, between .05 and .08 is seen as indicating a good fit, between .08 to .10 shows a poor fit, and over .10 suggests a bad fit (Byrne, 2012). A CFI [34] and TLI [35] > .90 are interpreted as indicating an acceptable fit.

We first tested the six-factor model, then the second-order hierarchical model and lastly the bi-factor model. As hypothesized because of the size of the sample, the chi-square of the six-factor model was significant, χ^2^(277) = 1372, p < .001. The χ^2^/df ratio was 4.95, which is considered to be acceptable [36]. For the other fit indices, we obtained an SRMR of .041 and a RMSEA of .050, a CFI of .94 and a TLI of .93. The combination of these different indices indicates a good fit. The standardized factor loadings for the twenty-six items of the scale can be found in S1 File.

We then conducted a CFA to assess if a single higher-order factor of self-compassion could explain the inter-correlations among the six factors as presented in the initial validation study [6]. As can be seen in Table 2, a single order model exhibited weaker fit indices: χ^2^(286) = 2284, p < .001. χ^2^/df = 7.98. The SRMR was .066 and the RMSEA was .065, and there was a CFI of .88 and a TLI of .89, with the latter two values being within the commonly accepted threshold. It has to be noted that similar results were found in the initial validation study [6], with the two only fit indicators reported in that study being an NNFI = .88 and a CFI = .90.

**Table 2:** CFA results for the 3 different models tested.

Model	χ²	Df	χ² / df	RMSEA [90%CI]	SRMR	TLI	CFI
Six-factor	1372	277	4.95	.050 [.047, .053]	.041	.93	.94
Hierarchical	2284	286	7.98	.067 [.065, .070]	.066	.88	.89
Bi-factor	1815	267	6.23	.058 [.056, .061]	.047	.91	.92

RMSEA = Root Mean Square Error of Approximation, [90%CI] = RMSEA Confidence Interval at 90%, SRMR = Standardized Root Mean Square Residual, TLI = Tucker-Lewis Index, CFI = Comparative Fit Index.

We lastly conducted a CFA to assess the bi-factor model and calculated the omega index. Fit indices of the bi-factor model, as reported in Table 2 were: χ^2^(291) = 1815, p < .001 and χ^2^/df = 6.23. The SRMR was .047, the RMSEA was .058, the CFI was .92 and the TLI was .91, with the combination of these different indices indicating an acceptable fit. The omega index was 0.94. Results are summarized in Table 2.

In order to evaluate the reliability of the French version of the SCS, internal consistency was assessed with Cronbach’s *α* coefficients [37]. Cronbach’s α range from .74 to .88. Thus, the factors of common humanity (.74), over-identified (.77) and isolation (.79) each had good level of reliability (> .70), while mindfulness (.81), self-judgment (.85) and self-kindness (.89) each had a very good level of reliability (> .80). Internal reliability for the total score was excellent (.94). Means and standard deviations for each of the subscales can be found in Table 3.

**Table 3:** Mean scores and Cronbach’s αs for each subscale.

Subscale	α	Mean
Self-Kindness	.88	14.27 (5.05)
Self-Judgment	.85	14.23 (5.03)
Common humanity	.74	11.56 (3.64)
Isolation	.79	11.84 (4.19)
Mindfulness	.81	13.81 (3.72)

### Correlations

The mean score for the SCS scale was 3.63 (SD = .92). Correlations between SCS and the other measures were computed. As expected, because they capture related constructs, self-compassion (SCS) and mindfulness (MAAS) were significantly inter-correlated. As predicted by previous studies [38], SCS also showed good convergent validity with well-being indicators as satisfaction with life (SWLS), and subjective happiness (SHS). As expected for the same reasons, the SCS scale also showed a positive correlation with positive affect (PA), a negative one with negative affect (NA) and a strong negative correlation with depression (BDI). Pearson’s correlations are reported in Table 4.

**Table 4:** Pearson’s correlations among all measures.

	SCS	MAAS	SHS	SWLS	NA	PA	BDI
SCS	1	.54***	.58***	.50***	-.63***	.48***	-.66***
MAAS		1	.40***	.35***	-.51***	.37***	-.52***
SHS			1	.63***	-.50***	.52***	-.65***
SWLS				1	-.45***	.49***	.65***
NA					1	-.26***	.62***
PA						1	-.53***
BDI							1

*** *p*.≤ 001.

### Application: Testing the moderation of SCS on the link between affect and depression

**Fig 1:**
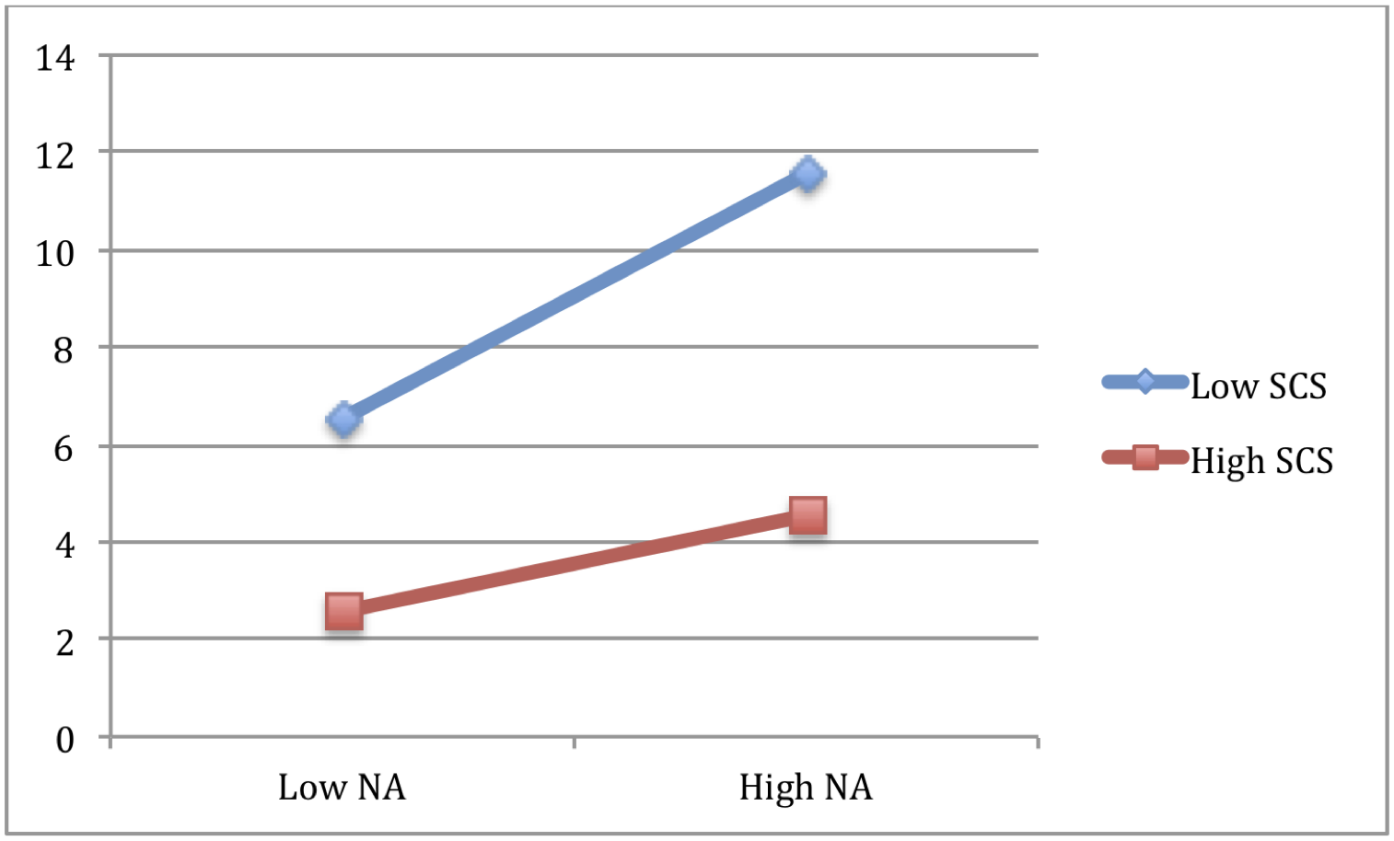
Moderation of SCS on the impact of NA on depression. Coordinates are 1 SD above and 1 SD below for both SCS and NA.

To test our hypothesis, we performed a moderation analysis between self-compassion and negative affect, and between self-compassion and positive affect, with BDI-SF (assessing depression symptoms) set as the outcome. Consistent with H1, a significant self-compassion x negative affect interaction (see Table 5) was found, indicating that at low levels of self-compassion, a high level of negative affect was associated with higher levels of depression, but that at high levels of self-compassion, it was associated with lower levels of depression. This suggests that SC protects against depression even in the presence of negative affect (see Fig 1).

**Table 5:** Model summary for the interaction of self-compassion with negative affect.

Model	Regression coefficients	Standard error	Bêta	p.	LLCI	ULCI
Intercept	6.30	.13		< .001	6.05	6.55
SCS	-3.47	.20	-.48	< .001	-3.86	-3.08
NA	.21	.02	.30	< .001	.17	.25
SCS x NA	-.11	.02	-.14	< .001	-.15	-.08

Note: R^2 = .53, F(3,1550) = 600, p. < .001.

Moreover, consistent with previous findings, our analyses revealed a significant main effect of negative affect, indicating that participants with higher levels of negative affect reported a higher level of depression. There was also a main effect of self-compassion, indicating that participants with greater self-compassion exhibited lower depression scores (see Table 5).

**Fig 2:**
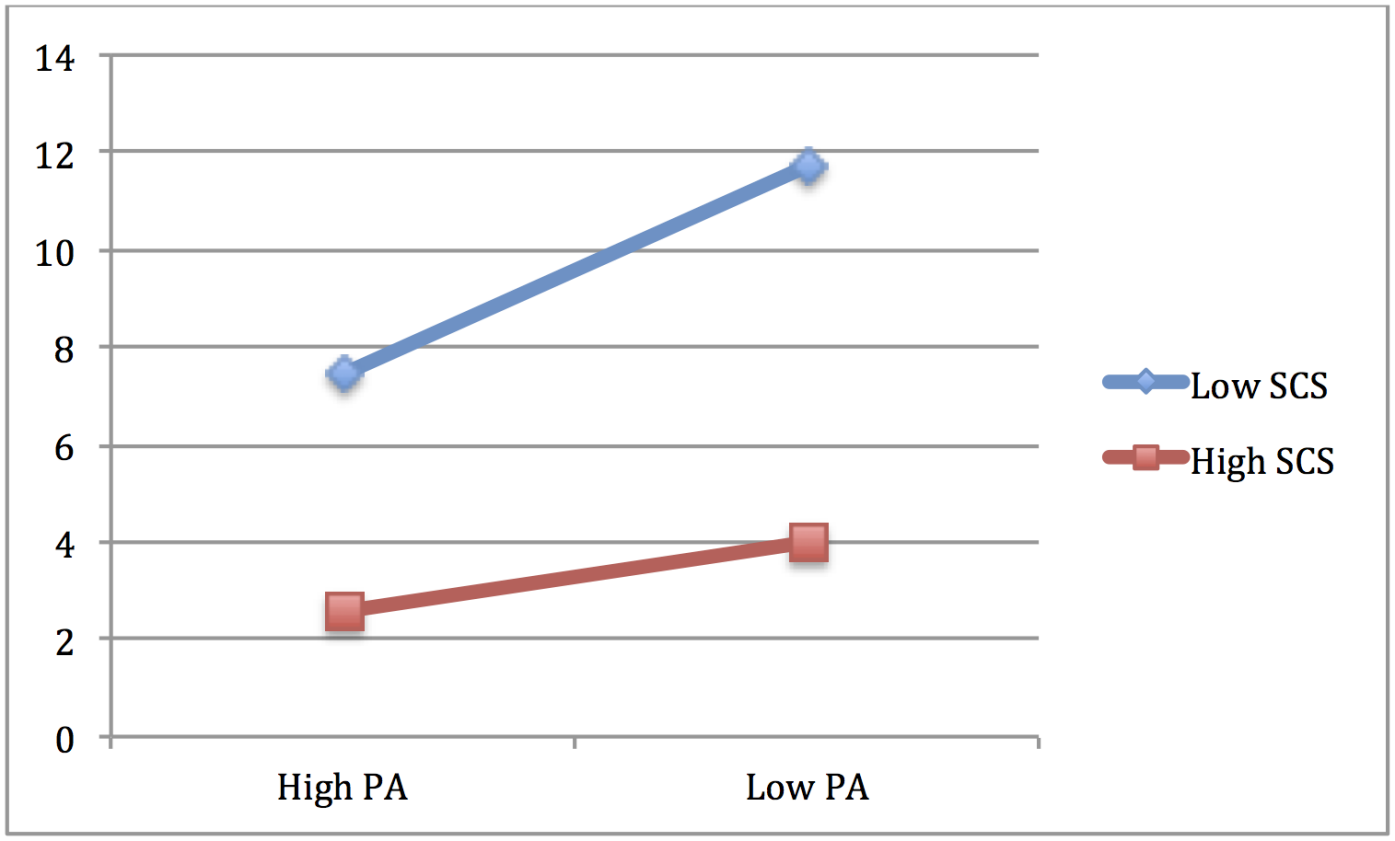
Moderation of SCS on the impact of PA on depression. Coordinates are 1 SD above and 1 SD below for both SCS and PA.

In line with H2, we found a significant interaction of self-compassion with positive affect, indicating that at low levels of self-compassion, a low level of positive affect is associated with higher levels of depression, but that a high level of self-compassion is associated with lower levels of depression even in absence of PA (see Table 6 and Fig 2).

**Table 6:** Model Summary for the interaction PA x SCS.

Model	Regression coefficients	Standard error	Bêta	P	LLCI	ULCI
Intercept	6.43	.12		< .001	6.20	6.66
SCS	-4.01	.17	-.54	< .001	-4.34	-3.68
PA	-.22	.02	-.27	< .001	-.26	-.18
SCS x PA	-.14	.02	-.18	< .001	.10	-.18

Note: R^2 = .52, F(3,1550) = 568, p. < .001.

Moreover, consistent with previous findings, analyses revealed a significant main effect of positive affect, indicating that participants with higher scores reported a lower level of depression. There was also a main effect of self-compassion, indicating that participants with greater self-compassion exhibited lower depression scores (see Table 6).

## Discussion

This study aimed: (a) to explore the factorial structure of the French version of the SCS, and (b) to examine the relationships among self-compassion and diverse indices of well-being (MAAS, SHS, SWLS), and (c) to asses the hypothesis that self-compassion acts as a moderator of the impact of affect (PANAS) on depression. The confirmatory factor analysis confirmed the 6-factor structure of the scale, while the fit indices for a possible second-order overarching factor were less satisfying. We also tested a bi-factor model and calculated an omega index, following the recommendation of recent research in the field [7]. The omega index was 0.94, indicating that the overall factor of self-compassion accounted for a large percentage of the variance, therefore justifying the use of an overall score [7].

The internal reliability scores of the SCS were found to be very good. The psychometric properties of the French version of the SCS are at least comparable to those of the original English-version of the scale [6]. Moreover, the convergent validity was satisfying, since it was shown, consistent with previous findings, that a high level of self-compassion is linked with higher attention and awareness (MAAS) and improved psychological well-being (happiness and life satisfaction). The present study is also consistent with earlier findings, showing a positive association between negative affect and depression [20] and a negative association between self-compassion and depression [12]

In line with Neff et al. (2007), we tested the hypothesis that self-compassion is a key mechanism accounting for the link between affect (both positive and negative) and depression symptoms. Our results are consistent with the idea that the harmful effect of negative affect is reduced when people are kind with themselves (i.e., have a high level of self-compassion). Conversely, the absence of positive affect is known to yield depressive symptoms (anhedony). However, results show that a high level of self-compassion can temper this effect. These results are in line with findings of contextual behavioral therapies [22] such as acceptance and commitment therapy [39] or dialectical behavior therapy [40], which postulate that the acceptance of private experiences as emotions can reduce reactivity to those private experiences and to the contexts that trigger them (Hayes et al., 2004).

### Limitations and future directions

There are a number of limitations to the current study. First and foremost, is important to note that this study investigated a non-clinical sample with low levels of depression. Further studies should assess the same hypothesis with a clinical sample. Second, due to the cross-sectional design, there is no certainty about the causality of the relationships. Future longitudinal studies may address this concern. Third, we focused on the link between affect and depression, but affect can also be seen as a byproduct of emotion regulation strategies. Future research may focus on the possible moderating role of self-compassion on the link between emotion-regulation strategies and depression.

In conclusion, this French version of the SCS is a valid tool that assesses individual differences in self-compassion. Because of the important consequences of self-compassion on well-being and psychological health, this scale allows the possibility of investigating these consequences in French-speaking populations. However, our study also highlights the need for further research in the field of self-compassion.

## Supporting Information

S1 File Factor pattern table. (DOCX)

S2 File French version of the SCS questionnaire. (PDF)

S3 File Correlation matrix. (DOCX)

S4 File Subscales correlation table. (DOCX)

S5 File Retrospective waivers of approval. (PDF)
