# Supplementary material for: Self-Compassion Scale (SCS): Psychometric Properties of The French Translation and Its Relations with Psychological Well-Being, Affect and Depression
Source: PLoS One. 2016 Apr 14;11(4):e0152880. doi: 10.1371/journal.pone.0152880 (PMC4831759; doi:10.1371/journal.pone.0152880)
Supplement: S1 File — (DOCX) [file pone.0152880.s001.docx]

**S1. Factor pattern table**

Standardized estimates for the different models.

6 factor model.

| F1 by | Estimate (S.E.) | F2 by | Estimate (S.E.) | F3 by | Estimate (S.E.) | F4 by | Estimate (S.E.) | F5 by | Estimate (S.E.) | F6 by | Estimate (S.E.) |
| --- | --- | --- | --- | --- | --- | --- | --- | --- | --- | --- | --- |
| Item 11 | 0.946 (0.025) | Item 15 | 0.951 (0.025) | Item 23 | 0.913 (0.025) | Item 12 | 0.997 (0.026) | Item 21 | 0.768 (0.024) | Item 13 | 0.983 (0.024) |
| Item 22 | 1.016 (0.022) | Item 5 | 0.840 (0.027) | Item 9 | 0.802 (0.026) | Item 6 | 0.924 (0.028) | Item 18 | 0.914 (0.022) | Item 8 | 0.932 (0.026) |
| Item 26 | 1.026 (0.022) | Item 25 | 0.992 (0.026) | Item 7 | 0.471 (0.037) | Item 4 | 0.779 (0.029) | Item 3 | 0.842 (0.024) | Item 1 | 0.699 (0.026) |
| Item 2 | 0.838 (0.025) | Item 14 | 0.945 (0.025) | Item 17 | 0.697 (0.030) | Item 24 | 0.822 (0.030) | Item 16 | 0.828 (0.026) | Item 19 | 0.780 (0.028) |
| Item 20 | 0.898 (0.024) | Item 10 | 0.814 (0.029) |  |  |  |  |  |  |  |  |

F1 = Self-Kindness, F2 = Self-Judgment, F3 = Common Humanity, F4 = Isolation, F5 = Mindfulness, F6 = Over-identification.

One factor second order model

| F0 by | Estimate (S.E.) |  |  |  |  |  |  |  |  |  |  |
| --- | --- | --- | --- | --- | --- | --- | --- | --- | --- | --- | --- |
| F1 | 0.878 (0.009) |  |  |  |  |  |  |  |  |  |  |
| F2 | 0.867 (0.009) |  |  |  |  |  |  |  |  |  |  |
| F3 | 0.808 (0.017) |  |  |  |  |  |  |  |  |  |  |
| F4 | 0.861 (0.012) |  |  |  |  |  |  |  |  |  |  |
| F5 | 0.889 (0.009) |  |  |  |  |  |  |  |  |  |  |
| F6 | 0.876 (0.010) |  |  |  |  |  |  |  |  |  |  |
| F1 by | Estimate (S.E.) | F2 by | Estimate (S.E.) | F3 by | Estimate (S.E.) | F4 by | Estimate (S.E.) | F5 by | Estimate (S.E.) | F6 by | Estimate (S.E.) |
| Item 11 | 0.960 (0.025) | Item 15 | 0.956 (0.025) | Item 23 | 0.928 (0.026) | Item 12 | 0.990 (0.026) | Item 21 | 0.778 (0.024) | Item 13 | 0.982 (0.025) |
| Item 22 | 1.040 (0.022) | Item 5 | 0.830 (0.027) | Item 9 | 0.793 (0.027) | Item 6 | 0.941 (0.029) | Item 18 | 0.920 (0.023) | Item 8 | 0.901 (0.028) |
| Item 26 | 1.047 (0.022) | Item 25 | 1.017 (0.027) | Item 7 | 0.464 (0.037) | Item 4 | 0.793 (0.030) | Item 3 | 0.850 (0.024) | Item 1 | 0.725 (0.027) |
| Item 2 | 0.820 (0.026) | Item 14 | 0.923 (0.026) | Item 17 | 0.688 (0.030) | Item 24 | 0.810 (0.032) | Item 16 | 0.793 (0.029) | Item 19 | 0.801 (0.028) |
| Item 20 | 0.869 (0.025) | Item 10 | 0.801 (0.030) |  |  |  |  |  |  |  |  |

F1 = Self-Kindness, F2 = Self-Judgment, F3 = Common Humanity, F4 = Isolation, F5 = Mindfulness, F6 = Over-identification.

Bi factor model.

| F1 by | Estimate (S.E.) |  | Estimate (S.E.) |
| --- | --- | --- | --- |
| Item 11 | 0.922 (0.025) | Item 15 | 0.915 (0.025) |
| Item 22 | 0.994 (0.022) | Item 5 | 0.805 (0.027) |
| Item 26 | 0.988 (0.022) | Item 25 | 0.934 (0.027) |
| Item 2 | 0.807 50.025) | Item 14 | 0.947 (0.024) |
| Item 20 | 0.882 (0.023) | Item 10 | 0.767 (0.029) |
| Item 23 | 0.824 (0 .025) | Item 12 | 0.966 (0.025) |
| Item 9 | 0.743 (0.026) | Item 6 | 0.893 (0.028) |
| Item 7 | 0.358 (0.036) | Item 4 | 0.742 (0.029) |
| Item 17 | 0.606 (0.029) | Item 24 | 0.800 (0.029) |
| Item 21 | 0.739 (0.024) | Item 13 | 0.952 (0.024) |
| Item 18 | 0.859 (0.023) | Item 8 | 0.938 (0.025) |
| Item 3 | 0.824 (0.024) | Item 1 | 0.664 (0.026) |
| Item 16 | 0.812 (0.026) | Item 19 | 0.737 (0.028) |

F1 = Self-Kindness, F2 = Self-Judgment, F3 = Common Humanity, F4 = Isolation, F5 = Mindfulness, F6 = Over-identification.
