# Supplementary material for: Self-Compassion Scale (SCS): Psychometric Properties of The French Translation and Its Relations with Psychological Well-Being, Affect and Depression
Source: PLoS One. 2016 Apr 14;11(4):e0152880. doi: 10.1371/journal.pone.0152880 (PMC4831759; doi:10.1371/journal.pone.0152880)
Supplement: S2 File — (PDF) [file pone.0152880.s002.pdf]

S2. French version of the SCS questionnaire.

Echelle d'auto-compassion (EAC)

Référence: Kotsou I, Leys C (2016) Self-Compassion Scale (SCS): Psychometric Properties of The French Translation and Its Relations with Psychological Well-Being, Affect and Depression. PLoS ONE.

Codage:

Items d'auto-bienveillance: 5, 12, 19, 23, 26

Items d'auto-jugement: 1, 8, 11, 16, 21

Items de commune humanité: 3, 7, 10, 15

Items d'isolation: 4, 13, 18, 25

Items de mindfulness: 9, 14, 17, 22

Items de sur-identification: 2, 6, 20, 24

Les scores des 6 sous-dimensions se calculent en faisant la moyenne des items de chaque sous-dimension.

Afin de calculer un score total d'auto-compassion, il convient de reverser le score des items des sous-dimensions "auto-jugement", "isolation" et "sur-identification". (i.e., 1 = 5, 2 = 4, 3 = 3. 4 = 2, 5 = 1) et ensuite calculer le score total.

Lisez attentivement chaque énoncé avant de répondre. A droite de chaque item, indiquez à quelle fréquence vous vous comportez de cette façon, en utilisant l'échelle de 1 à 5.

1	2	3	4	5
**Presque jamais**				**Presque toujours**

1.	Je désapprouve et juge mes propres défauts et insuffisances.	
2.	Lorsque je me sens mal, j'ai tendance à être obsédé(e) et à focaliser sur tout ce qui ne va pas.	
3.	Quand les choses vont mal pour moi, je vois ces difficultés comme faisant partie de la vie que chacun traverse.	
4.	Quand je pense à mes insuffisances, je me sens différent(e) et coupé(e) du reste du monde.	
5.	J'essaye d'être aimant(e) envers moi-même quand je souffre.	
6.	Quand j'échoue à quelque chose d'important pour moi, je suis envahi(e) par un sentiment de ne pas être à la hauteur.	
7.	Quand je me sens déprimé(e), je me rappelle qu'il y a beaucoup d'autres personnes dans le monde qui ressentent la même chose.	
8.	Quand les choses vont vraiment mal, j'ai tendance à être dur(e) envers moi-même	
9.	Quand quelque chose me contrarie, j'essaye de garder mes émotions en équilibre.	
10.	Quand je ne me sens pas à la hauteur d'une quelconque façon, j'essaye de me rappeler que ce sentiment est partagé par la plupart des gens.	
11.	Je suis intolérant(e) et impatient(e) envers les aspects de ma personnalité que je n'aime pas.	
12.	Quand je traverse une période très difficile, je me donne le soin et la tendresse dont j'ai besoin.	

13.	Quand je me sens mal, j'ai tendance à avoir l'impression que les autres sont plus heureux que moi.	
14.	Quand quelque chose de douloureux se produit, j'essaye d'avoir une vision équilibrée de la situation.	
15.	J'essaye de voir mes défauts comme faisant partie de la condition humaine.	
16.	Quand je vois des aspects de moi-même que je n'aime pas, je me critique.	
17.	Quand j'échoue à quelque chose d'important pour moi j'essaye de garder les choses en perspective.	
18.	Quand c'est vraiment difficile pour moi, j'ai tendance à penser que la vie est plus facile pour les autres.	
19.	Je suis bienveillant(e) envers moi-même quand je souffre	
20.	Quand quelque chose me perturbe, je me laisse emporter par mes sentiments.	
21.	Je suis dur(e) envers moi-même quand je ressens de la souffrance.	
22.	Quand je suis déprimé(e), je cherche à approcher mes sentiments avec curiosité et ouverture.	
23.	Je suis tolérant(e) avec mes propres défauts et insuffisances	
24.	Quand quelque chose de douloureux se produit, j'ai tendance à donner une importance hors de proportion à l'incident.	
25.	Quand j'échoue à quelque chose d'important pour moi, j'ai tendance à me sentir seul(e) dans mon échec.	
26.	J'essaye d'être compréhensif(ve) et patient(e) envers les aspects de ma personnalité que je n'aime pas.	
