# Supplementary material for: Self-Compassion Scale (SCS): Psychometric Properties of The French Translation and Its Relations with Psychological Well-Being, Affect and Depression
Source: PLoS One. 2016 Apr 14;11(4):e0152880. doi: 10.1371/journal.pone.0152880 (PMC4831759; doi:10.1371/journal.pone.0152880)
Supplement: S3 File — (DOCX) [file pone.0152880.s003.docx]

**S3. Correlation matrix**

Corrrelation matrix. Diagonal = variance.

|  | 1 | 2 | 3 | 4 | 5 | 6 | 7 | 8 | 9 | 10 | 11 | 12 | 13 | 14 | 15 | 16 | 17 | 18 | 19 | 20 | 21 | 22 | 23 | 24 | 25 | 26 |
| --- | --- | --- | --- | --- | --- | --- | --- | --- | --- | --- | --- | --- | --- | --- | --- | --- | --- | --- | --- | --- | --- | --- | --- | --- | --- | --- |
| 1 | 1.333 |  |  |  |  |  |  |  |  |  |  |  |  |  |  |  |  |  |  |  |  |  |  |  |  |  |
| 2 | 0.302 | 1.421 |  |  |  |  |  |  |  |  |  |  |  |  |  |  |  |  |  |  |  |  |  |  |  |  |
| 3 | 0.337 | 0.553 | 1.342 |  |  |  |  |  |  |  |  |  |  |  |  |  |  |  |  |  |  |  |  |  |  |  |
| 4 | 0.335 | 0.291 | 0.364 | 1.749 |  |  |  |  |  |  |  |  |  |  |  |  |  |  |  |  |  |  |  |  |  |  |
| 5 | 0.356 | 0.471 | 0.363 | 0.370 | 1.501 |  |  |  |  |  |  |  |  |  |  |  |  |  |  |  |  |  |  |  |  |  |
| 6 | 0.390 | 0.357 | 0.395 | 0.714 | 0.445 | 1.921 |  |  |  |  |  |  |  |  |  |  |  |  |  |  |  |  |  |  |  |  |
| 7 | 0.077 | 0.132 | 0.181 | 0.049 | 0.051 | 0.058 | 1.677 |  |  |  |  |  |  |  |  |  |  |  |  |  |  |  |  |  |  |  |
| 8 | 0.408 | 0.431 | 0.416 | 0.419 | 0.516 | 0.472 | 0.041 | 1.682 |  |  |  |  |  |  |  |  |  |  |  |  |  |  |  |  |  |  |
| 9 | 0.242 | 0.506 | 0.458 | 0.227 | 0.323 | 0.250 | 0.290 | 0.308 | 1.353 |  |  |  |  |  |  |  |  |  |  |  |  |  |  |  |  |  |
| 10 | 0.290 | 0.376 | 0.333 | 0.299 | 0.610 | 0.370 | 0.019 | 0.464 | 0.246 | 1.647 |  |  |  |  |  |  |  |  |  |  |  |  |  |  |  |  |
| 11 | 0.266 | 0.493 | 0.504 | 0.269 | 0.388 | 0.335 | 0.192 | 0.344 | 0.465 | 0.369 | 1.547 |  |  |  |  |  |  |  |  |  |  |  |  |  |  |  |
| 12 | 0.365 | 0.392 | 0.390 | 0.437 | 0.437 | 0.491 | 0.087 | 0.545 | 0.281 | 0.436 | 0.311 | 1.856 |  |  |  |  |  |  |  |  |  |  |  |  |  |  |
| 13 | 0.492 | 0.361 | 0.408 | 0.462 | 0.427 | 0.515 | 0.079 | 0.529 | 0.267 | 0.378 | 0.370 | 0.440 | 1.832 |  |  |  |  |  |  |  |  |  |  |  |  |  |
| 14 | 0.411 | 0.436 | 0.392 | 0.398 | 0.459 | 0.473 | 0.022 | 0.536 | 0.271 | 0.437 | 0.366 | 0.544 | 0.615 | 1.618 |  |  |  |  |  |  |  |  |  |  |  |  |
| 15 | 0.394 | 0.508 | 0.388 | 0.344 | 0.528 | 0.413 | 0.043 | 0.525 | 0.332 | 0.473 | 0.363 | 0.511 | 0.530 | 0.684 | 1.560 |  |  |  |  |  |  |  |  |  |  |  |
| 16 | 0.246 | 0.379 | 0.455 | 0.244 | 0.270 | 0.307 | 0.234 | 0.270 | 0.403 | 0.207 | 0.492 | 0.242 | 0.319 | 0.277 | 0.237 | 1.568 |  |  |  |  |  |  |  |  |  |  |
| 17 | 0.182 | 0.281 | 0.320 | 0.136 | 0.209 | 0.198 | 0.515 | 0.227 | 0.431 | 0.145 | 0.361 | 0.212 | 0.220 | 0.176 | 0.192 | 0.438 | 1.374 |  |  |  |  |  |  |  |  |  |
| 18 | 0.365 | 0.434 | 0.563 | 0.366 | 0.323 | 0.402 | 0.228 | 0.354 | 0.441 | 0.267 | 0.501 | 0.338 | 0.440 | 0.383 | 0.344 | 0.544 | 0.406 | 1.371 |  |  |  |  |  |  |  |  |
| 19 | 0.469 | 0.292 | 0.370 | 0.381 | 0.327 | 0.393 | 0.091 | 0.379 | 0.278 | 0.329 | 0.282 | 0.376 | 0.506 | 0.396 | 0.392 | 0.235 | 0.179 | 0.418 | 1.612 |  |  |  |  |  |  |  |
| 20 | 0.288 | 0.630 | 0.546 | 0.278 | 0.436 | 0.314 | 0.195 | 0.391 | 0.554 | 0.385 | 0.578 | 0.361 | 0.370 | 0.427 | 0.438 | 0.489 | 0.376 | 0.541 | 0.303 | 1.303 |  |  |  |  |  |  |
| 21 | 0.441 | 0.434 | 0.491 | 0.319 | 0.324 | 0.351 | 0.171 | 0.352 | 0.402 | 0.309 | 0.460 | 0.345 | 0.383 | 0.383 | 0.357 | 0.416 | 0.314 | 0.591 | 0.393 | 0.535 | 1.196 |  |  |  |  |  |
| 22 | 0.359 | 0.523 | 0.521 | 0.291 | 0.438 | 0.368 | 0.185 | 0.412 | 0.448 | 0.395 | 0.666 | 0.368 | 0.427 | 0.416 | 0.407 | 0.541 | 0.399 | 0.548 | 0.320 | 0.611 | 0.537 | 1.573 |  |  |  |  |
| 23 | 0.296 | 0.408 | 0.482 | 0.337 | 0.312 | 0.374 | 0.336 | 0.331 | 0.490 | 0.257 | 0.439 | 0.299 | 0.382 | 0.342 | 0.326 | 0.443 | 0.468 | 0.557 | 0.328 | 0.498 | 0.462 | 0.558 | 1.463 |  |  |  |
| 24 | 0.349 | 0.317 | 0.345 | 0.376 | 0.409 | 0.403 | 0.079 | 0.482 | 0.258 | 0.342 | 0.291 | 0.481 | 0.461 | 0.442 | 0.438 | 0.209 | 0.209 | 0.271 | 0.357 | 0.268 | 0.277 | 0.357 | 0.264 | 1.637 |  |  |
| 25 | 0.367 | 0.536 | 0.426 | 0.336 | 0.521 | 0.401 | 0.081 | 0.491 | 0.361 | 0.505 | 0.427 | 0.494 | 0.367 | 0.557 | 0.630 | 0.299 | 0.231 | 0.373 | 0.393 | 0.501 | 0.384 | 0.470 | 0.365 | 0.487 | 1.670 |  |
| 26 | 0.329 | 0.551 | 0.541 | 0.308 | 0.462 | 0.373 | 0.182 | 0.417 | 0.476 | 0.464 | 0.688 | 0.395 | 0.404 | 0.443 | 0.441 | 0.513 | 0.391 | 0.540 | 0.317 | 0.641 | 0.506 | 0.744 | 0.521 | 0.355 | 0.524 | 1.533 |
