# Supplementary material for: Self-Compassion Scale (SCS): Psychometric Properties of The French Translation and Its Relations with Psychological Well-Being, Affect and Depression
Source: PLoS One. 2016 Apr 14;11(4):e0152880. doi: 10.1371/journal.pone.0152880 (PMC4831759; doi:10.1371/journal.pone.0152880)
Supplement: S4 File — (DOCX) [file pone.0152880.s004.docx]

**S4. Subscales correlations table**

Table 4. Pearson’s correlations among the 6 subscales

|  | Self-Kindness | Self-Judgment | Common Humanity | Isolation | Mindfulness | Over-identification |
| --- | --- | --- | --- | --- | --- | --- |
| Self-Kindness | 1 | .66*** | .60*** | .51*** | .77*** | .55*** |
| Self-Judgment |  | 1 | .35*** | .67*** | .52*** | .70*** |
| Common Humanity |  |  | 1 | .35*** | .62*** | .38*** |
| Isolation |  |  |  | 1 | .52*** | .71*** |
| Mindfulness |  |  |  |  | 1 | .58*** |
| Over-identification |  |  |  |  |  | 1 |

*** *p*.≤ 001
