# Supplementary material for: Self-Compassion Scale (SCS): Psychometric Properties of The French Translation and Its Relations with Psychological Well-Being, Affect and Depression
Source: PLoS One. 2016 Apr 14;11(4):e0152880. doi: 10.1371/journal.pone.0152880 (PMC4831759; doi:10.1371/journal.pone.0152880)
Supplement: S5 File — (PDF) [file pone.0152880.s005.pdf]

ULB

UNIVERSITÉ LIBRE DE BRUXELLES, UNIVERSITÉ D'EUROPE

**COMITE D'ETHIQUE DE LA FACULTE DES SCIENCES PSYCHOLOGIQUES ET DE L'EDUCATION**
**Président : P. Peigneux**
**Secrétaire : O. Klein**
**Membres effectifs : A Bazan, B Dan, V Carette, C Hellemans, C Leys,**
**Membres suppléants : C Colin, S Kahn, L Licata, I Merckaert, C Mottrie, S Pohl**
Adresse de contact :
Prof P. Peigneux, CP191, Avenue F. D. Roosevelt 50, B-1050 Bruxelles (Belgique)
**T**el +32 (2) 650 26 39 (secrétariat 4581)
**F**ax +32 (2) 650 22 09
Email : Philippe.Peigneux@ulb.ac.be

Bruxelles, 21 of August, 2015

**Object: Retrospective waivers of approval**

I hereby confirms that questionnaire-based and survey studies such as described in the article "Self-Compassion Scale (SCS): Psychometric Properties of The French Translation and Its Relations with Psychological Well-Being, Affect and Depression", submitted for publication by Mr. Ilios Kotsou, were waived from the obligation to obtain the approval from our Faculty Ethics Committee at the time of data collection.

On behalf of the Ethics Committee of the Faculty of Psychological Sciences at the Université Libre de Bruxelles,

Prof Philippe Peigneux, President
